# 2-Chloro-3-nitro-5-(tri­fluoro­meth­yl)benzoic acid and -benzamide: structural characterization of two precursors for anti­tubercular benzo­thia­zinones

**DOI:** 10.1107/S2056989021000517

**Published:** 2021-01-19

**Authors:** Adrian Richter, Richard Goddard, Tom Schlegel, Peter Imming, Rüdiger W. Seidel

**Affiliations:** a Institut für Pharmazie, Martin-Luther-Universität Halle-Wittenberg, Wolfgang-Langenbeck-Str. 4, 06120 Halle (Saale), Germany; b Max-Planck-Institut für Kohlenforschung, Kaiser-Wilhelm-Platz 1, 45470 Mülheim an der Ruhr, Germany

**Keywords:** benzo­thia­zinones, BTZ043, PBTZ169, nitro­benzoic acids, nitro­benzamides, anti­tuberculosis drugs, hydrogen bonds, crystal structure

## Abstract

8-Nitro-1,3-benzo­thia­zin-4-ones are a promising class of new anti­tubercular agents, two candidates of which, namely BTZ043 and PBTZ169 (INN: macozinone), have reached clinical trials. The crystal and mol­ecular structures of two synthetic precursors, 2-chloro-3-nitro-5-(tri­fluoro­meth­yl)benzoic acid, C_8_H_3_ClF_3_NO_4_ (**1**), and 2-chloro-3-nitro-5-(tri­fluoro­meth­yl)benzamide, C_8_H_4_ClF_3_N_2_O_3_ (**2**), are reported. In **1** and **2**, the respective carb­oxy, carboxamide and the nitro groups are significantly twisted out of the plane of the benzene ring. In **1**, the nitro group is oriented almost perpendicular to the benzene ring plane. In the crystal, **1** and **2** form O—H⋯O and N—H⋯O hydrogen-bonded dimers, respectively, which in **2** extend into primary amide tapes along the [101] direction. The tri­fluoro­methyl group in **2** exhibits rotational disorder with an occupancy ratio of 0.876 (3):0.124 (3).

## Abstract 2

The crystal and mol­ecular structures of 2-chloro-3-nitro-5-(tri­fluoro­meth­yl)benzoic acid and 2-chloro-3-nitro-5-(tri­fluoro­meth­yl)benzamide, two precursors for the synthesis of 8-nitro-1,3-benzo­thia­zin-4-ones, a promising class of new anti­tuberculosis drug candidates, are described.

## Chemical context   

2-Chloro-3-nitro-5-(tri­fluoro­meth­yl)benzoic acid (**1**) and 2-chloro-3-nitro-5-(tri­fluoro­meth­yl)benzamide (**2**), the title compounds, have been used as precursors in various synthetic routes to 8-nitro-6-(tri­fluoro­meth­yl)benzo-1,3-thia­zin-4-ones (BTZ) (Makarov *et al.*, 2007 ▸; Moellmann *et al.*, 2009 ▸; Cooper *et al.*, 2011 ▸; Gao *et al.*, 2013 ▸; Rudolph, 2014 ▸; Peng *et al.*, 2015 ▸; Rudolph *et al.*, 2016 ▸; Zhang & Aldrich, 2019 ▸; Zhang *et al.*, 2019 ▸), a promising new class of anti­tubercular agents, which target the mycobacterial enzyme deca­prenyl­phosphoryl-β-d-ribose 2′-epimerase (DprE1) (Trefzer *et al.*, 2010 ▸, 2012 ▸; Mikušová *et al.*, 2014 ▸; Piton *et al.*, 2017 ▸; Richter *et al.*, 2018 ▸). Two compounds from this class, *viz.* BTZ043 and PBTZ169 (INN: macozinone), have already reached clinical trials (Makarov & Mikušová, 2020 ▸; Mariandyshev *et al.*, 2020 ▸; Shetye *et al.*, 2020 ▸).
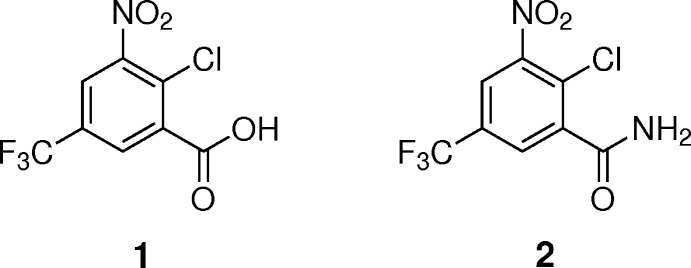


**Figure 1:**
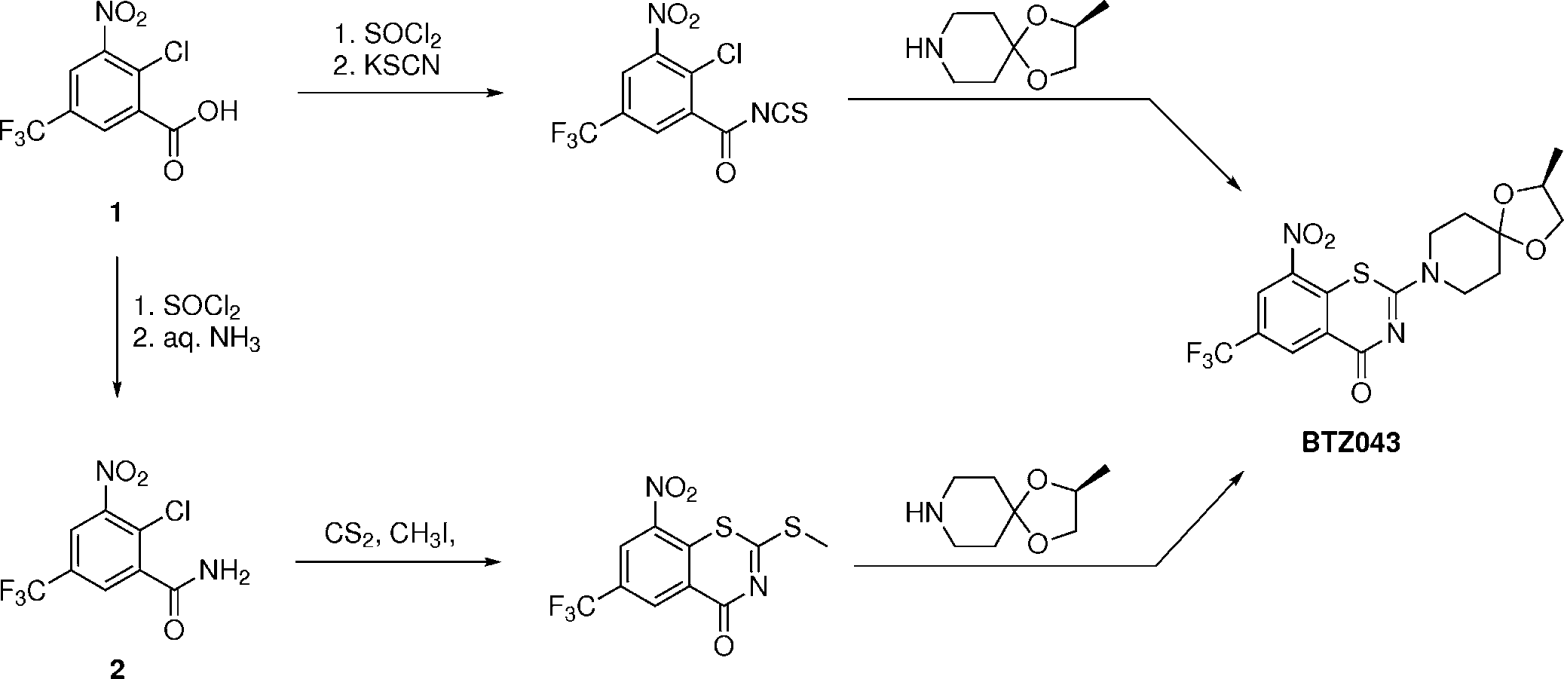
Conversion of **1** to **2** in two steps and schematic illustration of two representative syntheses of BTZ043 starting from **1** (Makarov *et al.*, 2007 ▸) or **2** (Makarov, 2011 ▸).

Fig. 1 ▸ depicts two representative syntheses of the lead compound BTZ043 starting from **1** or **2**. In the original synthesis, reaction of **1** (Makarov *et al.*, 2007 ▸) with potassium thio­cyanate after activation with thionyl chloride gives the highly reactive benzoyl iso­thio­cyanate derivative, which is reacted *in situ* with the secondary amine (*S*)-2-methyl-1,4-dioxa-8-aza­spiro­[4.5]decane to form a thio­urea derivative (not shown), which undergoes ring closure to form BTZ043. Starting from **2** (Makarov, 2011 ▸), reaction with carbon di­sulfide and methyl iodide leads to the stable 2-(methyl­thio)-8-nitro-6-(tri­fluoro­meth­yl)benzo-1,3-thia­zin-4-one. Reaction with the aforementioned secondary amine eventually affords BTZ043.

To the best of our knowledge, Welch *et al.* (1969 ▸) were the first to report the synthesis of the title compounds more than 50 years ago in the course of a study on tri­fluoro­methyl­benzamides as anti­coccidial agents. Compound **1** is readily obtained from 2-chloro-5-(tri­fluoro­meth­yl)benzo­nitrile upon reaction with nitrating acid mixture. Treatment of **1** with thionyl chloride affords the corresponding acid chloride, which is reacted with concentrated ammonia solution to give amide **2** in good yield (Fig. 1 ▸).

## Structural commentary   

**Figure 2:**
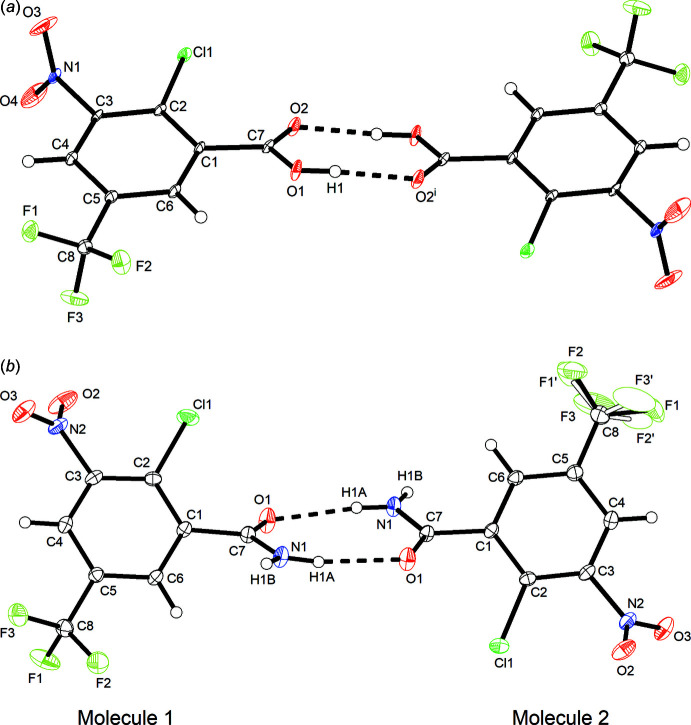
Hydrogen-bonded dimers of **1** (*a*) and **2** (*b*) in the crystal. Displacement ellipsoids are drawn at the 50% probability level. The site of the disordered tri­fluoro­methyl group in **2** with minor occupancy (*ca* 12%) in the crystal is shown by empty ellipsoids. Hydrogen atoms are represented by small spheres of arbitrary radius and hydrogen bonds are shown by dashed lines. Symmetry code: (i) −*x* + 2, −*y* + 1, −*z* + 1.

Fig. 2 ▸ shows the mol­ecular structures of **1** and **2** in the crystal. Both compounds form hydrogen-bonded dimers in the solid state, which in the case of **2** is augmented by additional N—H⋯O hydrogen bonds to form a catemer (see Section 3). In **1**, the plane defined by the carb­oxy group non-hydrogen atoms (O1, O2 and C7) is twisted out of the mean plane of the benzene ring (C1–C6) by 22.9 (1)°. Remarkably, the plane defined by the nitro group (O3, O4 and N1) is oriented nearly perpendicular to the mean plane of the benzene ring with a tilt angle of 85.38 (7)°.

Compound **2** crystallizes with two mol­ecules in the asymmetric unit (*Z*′ = 2), one of which exhibits partial rotational disorder of the tri­fluoro­methyl group. With respect to the mean plane of benzene ring (C1–C6), the plane defined by the non-hydrogen atoms of the amide group (O1, N1 and C7) is inclined at 49.0 (2) and 43.4 (2)° in mol­ecule 1 and 2, respectively. The tilt angle between the plane of the nitro group (O2, O3 and N2) and the benzene ring mean plane is 46.1 (1)° in mol­ecule 1 and 46.7 (1)° in mol­ecule 2, which is significantly smaller than in **1**.

The ^1^H NMR spectrum of **2** in DMSO-*d*_6_ at room temperature shows two distinct broad singlets for the amide hydrogen atoms (see supporting information), indicating restricted rotation about the C—N bond due to partial double-bond character (Wiberg, 2003 ▸). In the IR spectrum of solid **2** (see supporting information), two characteristic N—H stretching bands at 3356 and 3178 cm^−1^ are present (Parker, 1971 ▸).

## Supra­molecular features   

**Figure 3:**
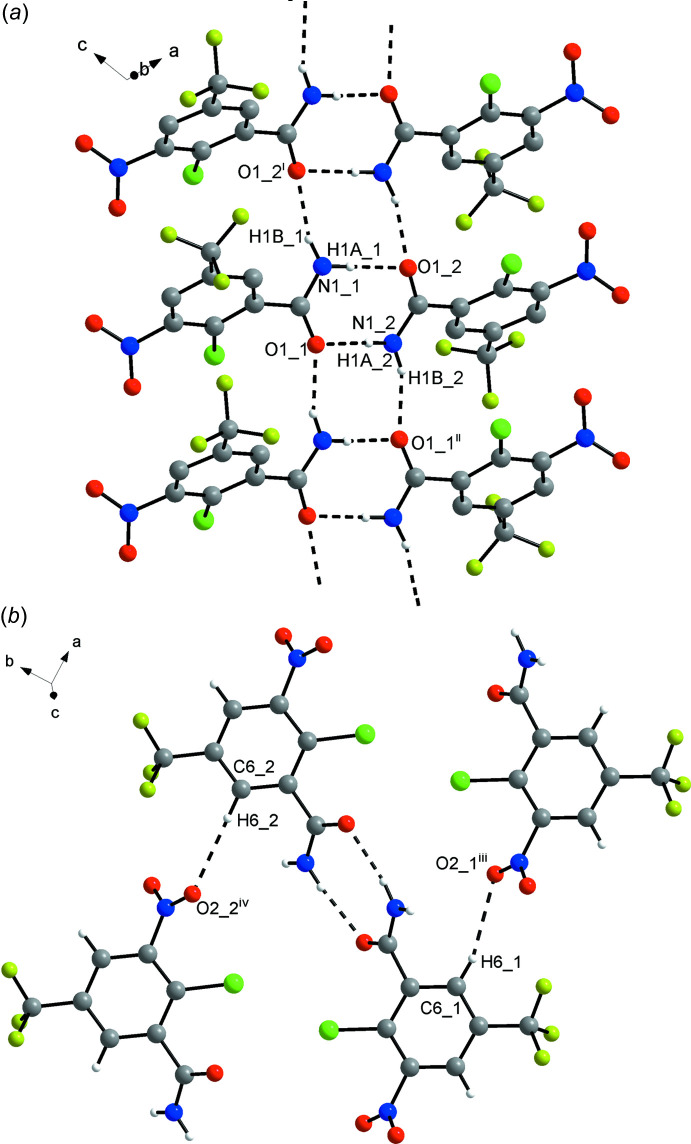
Parts of the crystal structure of **2** viewed (*a*) down [1
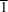
0], showing N—H⋯O hydrogen-bonded tapes extending in the [101] direction, and (*b*) towards the plane (001), showing short C—H⋯O contacts in addition to classical N—H⋯O hydrogen bonds (both represented by dashed lines). The number after the underscore indicate unique mol­ecule 1 or 2 (Fig. 2 ▸). The minor disorder part of the tri­fluoro­methyl group in mol­ecule 2 and carbon-bound hydrogen atoms are omitted for clarity in (*a*). Symmetry codes: (i) −*x* + 2, −*y* + 1; (ii) −*x* + 1, −*y* + 1, -*z;* (iii) *x* + 1, *y*, *z*; (iv) *x* − 1, *y*, *z*.

The supra­molecular structures of **1** and **2** feature carb­oxy­lic acid–carb­oxy­lic acid and amide–amide homosynthons (Desiraju, 1995 ▸; Thakuria *et al.*, 2017 ▸), respectively. The hydrogen-bond motif is 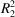
(8) (Bernstein *et al.*, 1995 ▸) in both cases. Geometric parameters of the O—H⋯O hydrogen bonds in **1** (Table 1 ▸) and the N—H⋯O hydrogen bonds in **2** (Table 2 ▸) are within expected ranges (Thakuria *et al.*, 2017 ▸). In **1** two mol­ecules related by crystallographic inversion symmetry form a carb­oxy­lic acid dimer, whereas in **2** two crystallographically unique mol­ecules related by approximate local inversion symmetry form a carboxamide dimer. The second amide hydrogen atom forms a hydrogen bond to the carbonyl oxygen atom of an adjacent dimer. The additional 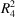
(8) hydrogen-bond motif thus formed about a crystallographic centre of symmetry extends the N—H⋯O hydrogen-bonding pattern in **2** into typical primary amide tapes (Leiserowitz & Schmidt, 1969 ▸) parallel to the [101] direction (Fig. 3 ▸ *a*).

In addition to classical O—H⋯O and N—H⋯O inter­molecular hydrogen bonds in **1** and **2**, respectively, the solid-state supra­molecular structures of both compounds feature a number of possible weak inter­actions (Tables 1 ▸ and 2 ▸). In **1** the C4—H4 moiety forms a short contact to a fluorine atom of the tri­fluoro­methyl group of a neighbouring mol­ecule (Fig. S1 in the supporting information) and the nitro group appears to accept a donating bifurcated weak C—H⋯O hydrogen bond from the C6—H6 moiety (Fig. S2 in the supporting information). The latter inter­action links the mol­ecules into chains in the [110] direction and may be discussed in connection with the remarkable twist of the nitro group out of the plane of the benzene ring. A packing index for **1** of 74.3%, as calculated with *PLATON* (Spek, 2020 ▸), indicates a fairly dense crystal packing for a mol­ecular compound (Kitaigorodskii, 1973 ▸).

In the crystal structure of **2**, short fluorine–fluorine contacts between the non-disordered tri­fluoro­methyl groups of adjacent mol­ecules 1 can be identified (Fig. S3 in the supporting information). Based on the corresponding C—F⋯F angles of 152.1 (1)° at F1 and 168.6 (1)° at F3, these contacts may be classified as type I fluorine–fluorine inter­actions (Baker *et al.*, 2012 ▸). As in **1**, the C6—H6 moieties in **2** form short C—H⋯O contacts to nitro oxygen atoms of adjacent mol­ecules (Fig. 3 ▸ *b*). The electron-withdrawing effect exerted by the ring subs­tituents in both **1** and **2** should activate the C—H moieties for weak hydrogen bonding (Thakuria *et al.*, 2017 ▸) to some extent. Notably, the packing index for **2** of 70.0%, as calculated only for the major disorder part of the tri­fluoro­methyl group in mol­ecule 2, is markedly smaller than for **1**.

## Database survey   

A search of the Cambridge Structural Database (CSD; version 5.41 with August 2020 updates; Groom *et al.*, 2016 ▸) for a 1-chloro-2-nitro-4-(tri­fluoro­meth­yl)benzene moiety revealed two related structures, *viz*. 1,5-di­chloro-2-nitro-4-(tri­fluoro­meth­yl)benzene (CSD refcode: JIHNOG) and 2-chloro-1,3-di­nitro-5-(tri­fluoro­meth­yl)benzene, also known as chloralin (JIHNUM) (del Casino *et al.*, 2018 ▸). In the two structures, the largest twist of a nitro group in *ortho* position to chlorine and *meta* position to the tri­fluoro­methyl group out of the benzene ring is 61.5° in JIHNUM. For [2-chloro-3-nitro-5-(tri­fluoro­meth­yl)phen­yl](piperidin-1-yl)methanone (MUPZAB), a structurally characterized side product in benzo­thia­zinone synthesis, the twist angle is 38.1 (2)° (Eckhardt *et al.*, 2020 ▸).

## Synthesis and crystallization   

*General:* Starting materials and reagents were obtained from chemical suppliers and used as received. Solvents were of reagent grade and were distilled before use. NMR spectra were measured on an Agilent Technologies VNMRS 400 MHz spectrometer. Chemical shifts are reported relative to the residual solvent peak of DMSO-*d*_6_ (δ_H_ = 2.50 ppm, δ_C_ = 39.5 ppm). Abbreviations: *s* = singlet, *d* = doublet, *q* = quartet. IR spectra were measured on a Bruker ALPHA Platinum ATR–FT–IR spectrometer. Mass spectra were recorded on a Thermo Fisher Q Exactive^TM^ Plus Orbitrap mass spectrometer for **1** and on an Advion expression^S^ compact mass spectrometer for **2**, using methanol as solvent.

*2-Chloro-3-nitro-5-(tri­fluoro­meth­yl)benzoic acid* (**1**): Compound **1** was synthesized from 2-chloro-5-(tri­fluoro­meth­yl)benzo­nitrile (Lundbeck) using a literature method (Welch *et al.*, 1969 ▸). ^1^H NMR (400 MHz, DMSO-*d*_6_): δ = 8.70 (*d*, *J* = 2.2 Hz, 1H), 8.40 (*d*, *J* = 2.2 Hz, 1H) ppm; ^13^C{^1^H} NMR (126 MHz, DMSO-*d*_6_) δ = 164.3, 149.7, 135.7, 129.7 (*q*, ^3^ *J* = 4 Hz), 128.8 (*q*, ^2^ *J* = 35 Hz), 127.2, 124.0 (*q*, ^3^ *J* = 4 Hz), 122.3 (*q*, ^1^ *J* = 273 Hz) ppm; IR(ATR): *ν*~ = 3097 (C—H stretch), 2865 (O—H stretch), 1702 (C=O stretch), 1542, 1323 (NO_2_ stretch), 1117 (C—F stretch) cm^−1^; MS (ESI^−^) *m*/*z* [*M* − COOH]^−^ calculated for C_7_H_2_ClF_3_NO_2_^−^ 224.0, found 224.0, [*M* − H]^−^ calculated for C_8_H_2_ClF_3_NO_4_^−^ 268.0, found 268.0.

*2-Chloro-3-nitro-5-(tri­fluoro­meth­yl)benzamide* (**2**): Compound **2** was prepared from **1** following a published procedure (Makarov *et al.*, 2007 ▸). ^1^H NMR (400 MHz, DMSO-*d*_6_): δ = 8.60 (*d*, *J* = 2.2 Hz, 1H), 8.22 (*s*, 1H), 8.18 (*d*, *J* = 2.2 Hz, 1H), 8.03 (*s*, 1H) ppm; ^13^C{^1^H} NMR (101 MHz, DMSO-*d*_6_) δ = 165.1, 148.9, 140.8, 128.8 (*q*, ^2^ *J* = 34 Hz), 128.2 (*q*, ^3^ *J* = 4 Hz), 125.8, 122.42 (*q*, ^1^ *J* = 273 Hz), 122.4 (*q*, ^3^ *J* = 4 Hz) ppm; IR(ATR): *ν*~ = 3356, 3178 (N—H stretch), 3096 (C—H stretch), 1659 (C=O stretch), 1629 (N—H bend), 1537, 1317 (NO_2_ stretch), 1116 (C—F stretch) cm^−1^; MS (APCI^+^) *m*/*z* [*M* + H]^+^ calculated for C_8_H_5_ClF_3_N_2_O_3_^+^ 269.0, found 268.9.

Crystals suitable for single-crystal X-ray diffraction were obtained by slow evaporation at room temperature of **1** in methanol/water and of **2** in ethanol.

## Refinement   

Crystal data, data collection and structure refinement details are summarized in Table 3 ▸. Diffraction data for **1** were measured at the P11 beamline at PETRA III at DESY (Meents *et al.*, 2013 ▸; Burkhardt *et al.*, 2016 ▸). Rotational disorder of a tri­fluoro­methyl group in **2** was refined using a split model with similar distance restraints on the 1,2- and 1,3-distances and equal atomic displacement parameters for opposite fluorine atoms belonging to different disorder sites. Refinement of the ratio of occupancies by means of a free variable resulted in 0.876 (3):0.124 (3). Carbon-bound H atoms were placed in geometrically calculated positions with C—H = 0.95 Å, and refined with the appropriate riding model and *U*_iso_(H) = 1.2 *U*_eq_(C). The carb­oxy hydrogen atom in **1** was located in a difference-Fourier map and refined freely. The amide H atoms in **2** were also located in difference-Fourier maps and refined semi-freely with the N—H distances restrained to a target value of 0.88 (2) Å and with *U*_iso_(H) = 1.2 *U*_eq_(N).

## Supplementary Material

Crystal structure: contains datablock(s) global, 1, 2. DOI: 10.1107/S2056989021000517/tx2035sup1.cif

Structure factors: contains datablock(s) 1. DOI: 10.1107/S2056989021000517/tx20351sup2.hkl

Structure factors: contains datablock(s) 2. DOI: 10.1107/S2056989021000517/tx20352sup3.hkl

Additional structure pictures, 1H and 13C NMR spectra, IR spectra and mass spectra. DOI: 10.1107/S2056989021000517/tx2035sup4.pdf

Supporting information file. DOI: 10.1107/S2056989021000517/tx20351sup5.cml

Supporting information file. DOI: 10.1107/S2056989021000517/tx20351sup6.cdx

Supporting information file. DOI: 10.1107/S2056989021000517/tx20352sup7.cml

Supporting information file. DOI: 10.1107/S2056989021000517/tx20352sup8.cdx

CCDC references: 2055889, 2055890

Additional supporting information:  crystallographic information; 3D view; checkCIF report

## Figures and Tables

**Table 1:** Hydrogen-bond geometry (Å, °) for **1** [Chem scheme1]

*D*—H⋯*A*	*D*—H	H⋯*A*	*D*⋯*A*	*D*—H⋯*A*
O1—H1⋯O2^i^	0.86 (2)	1.83 (2)	2.6891 (13)	176 (2)
C4—H4⋯F3^ii^	0.95	2.66	3.5767 (16)	161
C6—H6⋯O3^iii^	0.95	2.66	3.5410 (19)	154
C6—H6⋯O4^iii^	0.95	2.62	3.5019 (17)	154

Symmetry codes: (i) 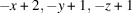; (ii) 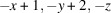; (iii) 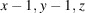.

**Table 2:** Hydrogen-bond geometry (Å, °) for **2** [Chem scheme1]

*D*—H⋯*A*	*D*—H	H⋯*A*	*D*⋯*A*	*D*—H⋯*A*
N1_1—H1*A*_1⋯O1_2	0.89 (2)	2.07 (2)	2.947 (2)	170 (2)
N1_1—H1*B*_1⋯O1_2^i^	0.89 (2)	1.99 (2)	2.840 (2)	160 (2)
N1_2—H1*A*_2⋯O1_1	0.86 (2)	2.08 (2)	2.940 (2)	172 (2)
N1_2—H1*B*_2⋯O1_1^ii^	0.88 (2)	1.99 (2)	2.804 (2)	153 (2)
C6_1—H6_1⋯O2_1^iii^	0.95	2.67	3.578 (2)	161
C6_2—H6_2⋯O2_2^iv^	0.95	2.48	3.430 (2)	179

Symmetry codes: (i) 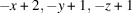; (ii) 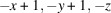; (iii) 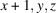; (iv) 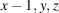.

**Table 3:** Experimental details

	**1**	**2**
Crystal data		
Chemical formula	C_8_H_3_ClF_3_NO_4_	C_8_H_4_ClF_3_N_2_O_3_
*M*_r_	269.56	268.58
Crystal system, space group	Triclinic, *P* 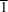	Monoclinic, *P*2_1_/*c*
Temperature (K)	100	100
*a*, *b*, *c* (Å)	4.7297 (10), 7.8993 (16), 13.044 (3)	8.3012 (12), 28.230 (4), 9.1522 (14)
α, β, γ (°)	91.57 (3), 96.51 (3), 104.79 (3)	90, 110.424 (3), 90
*V* (Å^3^)	467.36 (18)	2009.9 (5)
*Z*	2	8
Radiation type	Synchrotron, λ = 0.6199 Å	Mo *K*α
μ (mm^−1^)	0.31	0.42
Crystal size (mm)	0.33 × 0.20 × 0.04	0.05 × 0.02 × 0.01

Data collection		
Diffractometer	P11 beamline at Petra III with Pilatus 6M detector (Kraft *et al.*, 2009 ▸)	Bruker Kappa Mach3 APEXII
Absorption correction	–	Gaussian (*SADABS*; Bruker, 2012 ▸)
*T*_min_, *T*_max_	–	0.989, 0.996
No. of measured, independent and observed [*I* > 2σ(*I*)] reflections	9797, 2834, 2766	64252, 8316, 5593
*R*_int_	0.019	0.080
(sin θ/λ)_max_ (Å^−1^)	0.730	0.793

Refinement		
*R*[*F* ^2^ > 2σ(*F* ^2^)], *wR*(*F* ^2^), *S*	0.030, 0.082, 1.05	0.056, 0.136, 1.04
No. of reflections	2834	8316
No. of parameters	158	329
No. of restraints	0	121
H-atom treatment	H atoms treated by a mixture of independent and constrained refinement	H atoms treated by a mixture of independent and constrained refinement
Δρ_max_, Δρ_min_ (e Å^−3^)	0.64, −0.43	0.79, −0.59

Computer programs: *P11 Crystallography Control* (Meents *et al.*, 2013 ▸), *APEX3* (Bruker, 2017 ▸), *XDS* (Kabsch, 2010 ▸), *SAINT* (Bruker, 2004 ▸), *SHELXT* (Sheldrick, 2015*a* ▸), *SHELXL* (Sheldrick, 2015*b* ▸), *DIAMOND* (Brandenburg, 2018 ▸), *enCIFer* (Allen *et al.*, 2004 ▸) and *publCIF* (Westrip, 2010 ▸).

## supplementary crystallographic information

### 2-Chloro-3-nitro-5-(trifluoromethyl)benzoic acid (1) Crystal data

**Table:**

C_8_H_3_ClF_3_NO_4_	*Z* = 2
*M**_r_* = 269.56	*F*(000) = 268
Triclinic, *P*1	*D*_x_ = 1.916 Mg m^−^^3^
*a* = 4.7297 (10) Å	Synchrotron radiation, λ = 0.6199 Å
*b* = 7.8993 (16) Å	Cell parameters from 9110 reflections
*c* = 13.044 (3) Å	θ = 1.4–26.9°
α = 91.57 (3)°	µ = 0.31 mm^−^^1^
β = 96.51 (3)°	*T* = 100 K
γ = 104.79 (3)°	Plate, colourless
*V* = 467.36 (18) Å^3^	0.33 × 0.20 × 0.04 mm

### 2-Chloro-3-nitro-5-(trifluoromethyl)benzoic acid (1) Data collection

**Table:**

P11 beamline at Petra III with Pilatus 6M detector (Kraft *et al.*, 2009) diffractometer	2766 reflections with *I* > 2σ(*I*)
Radiation source: synchrotron	*R*_int_ = 0.019
Detector resolution: 5.81 pixels mm^-1^	θ_max_ = 26.9°, θ_min_ = 1.4°
φ scan	*h* = −6→6
9797 measured reflections	*k* = −11→11
2834 independent reflections	*l* = −19→19

### 2-Chloro-3-nitro-5-(trifluoromethyl)benzoic acid (1) Refinement

**Table:**

Refinement on *F*^2^	Primary atom site location: dual
Least-squares matrix: full	Secondary atom site location: difference Fourier map
*R*[*F*^2^ > 2σ(*F*^2^)] = 0.030	Hydrogen site location: mixed
*wR*(*F*^2^) = 0.082	H atoms treated by a mixture of independent and constrained refinement
*S* = 1.05	*w* = 1/[σ^2^(*F*_o_^2^) + (0.0446*P*)^2^ + 0.2681*P*] where *P* = (*F*_o_^2^ + 2*F*_c_^2^)/3
2834 reflections	(Δ/σ)_max_ = 0.001
158 parameters	Δρ_max_ = 0.64 e Å^−^^3^
0 restraints	Δρ_min_ = −0.43 e Å^−^^3^

### 2-Chloro-3-nitro-5-(trifluoromethyl)benzoic acid (1) Special details

**Table:**

Geometry. All esds (except the esd in the dihedral angle between two l.s. planes) are estimated using the full covariance matrix. The cell esds are taken into account individually in the estimation of esds in distances, angles and torsion angles; correlations between esds in cell parameters are only used when they are defined by crystal symmetry. An approximate (isotropic) treatment of cell esds is used for estimating esds involving l.s. planes.

### 2-Chloro-3-nitro-5-(trifluoromethyl)benzoic acid (1) Fractional atomic coordinates and isotropic or equivalent isotropic displacement parameters (Å^2^)

**Table:**

	*x*	*y*	*z*	*U*_iso_*/*U*_eq_	
C1	0.8267 (2)	0.79164 (11)	0.32984 (7)	0.00863 (16)	
C2	0.9897 (2)	0.96649 (11)	0.32560 (7)	0.00871 (16)	
C3	0.8979 (2)	1.06490 (11)	0.24763 (7)	0.00957 (17)	
C4	0.6603 (2)	0.99896 (12)	0.17358 (7)	0.01093 (17)	
H4	0.604630	1.069994	0.121438	0.013*	
C5	0.5045 (2)	0.82466 (12)	0.17771 (7)	0.00969 (16)	
C6	0.5845 (2)	0.72297 (11)	0.25554 (7)	0.01008 (17)	
H6	0.472768	0.605062	0.258199	0.012*	
C7	0.9092 (2)	0.67213 (11)	0.40892 (7)	0.00943 (17)	
C8	0.2606 (2)	0.74456 (13)	0.09293 (8)	0.01313 (18)	
N1	1.0646 (2)	1.24888 (10)	0.24249 (7)	0.01242 (16)	
O1	0.68411 (17)	0.53906 (9)	0.42044 (6)	0.01328 (15)	
H1	0.743 (5)	0.469 (3)	0.4618 (17)	0.037 (5)*	
O2	1.15606 (18)	0.69465 (9)	0.45466 (6)	0.01391 (15)	
O3	0.9986 (3)	1.35882 (11)	0.29446 (9)	0.0333 (3)	
O4	1.2576 (2)	1.27866 (12)	0.18631 (9)	0.0296 (2)	
F1	0.12540 (18)	0.86369 (9)	0.05423 (6)	0.02163 (16)	
F2	0.05311 (18)	0.61547 (10)	0.12436 (6)	0.02413 (17)	
F3	0.36346 (19)	0.68013 (11)	0.01315 (6)	0.02649 (18)	
Cl1	1.29187 (5)	1.06719 (3)	0.41157 (2)	0.01187 (7)	

### 2-Chloro-3-nitro-5-(trifluoromethyl)benzoic acid (1) Atomic displacement parameters (Å^2^)

**Table:**

	*U*^11^	*U*^22^	*U*^33^	*U*^12^	*U*^13^	*U*^23^
C1	0.0110 (4)	0.0033 (3)	0.0108 (4)	0.0005 (3)	0.0007 (3)	0.0019 (3)
C2	0.0106 (4)	0.0033 (3)	0.0106 (4)	−0.0007 (3)	0.0001 (3)	0.0003 (3)
C3	0.0133 (5)	0.0016 (3)	0.0122 (4)	−0.0008 (3)	0.0014 (3)	0.0015 (3)
C4	0.0149 (5)	0.0052 (3)	0.0114 (4)	0.0007 (3)	0.0003 (3)	0.0020 (3)
C5	0.0114 (4)	0.0053 (3)	0.0103 (4)	−0.0006 (3)	−0.0008 (3)	0.0004 (3)
C6	0.0125 (5)	0.0037 (3)	0.0124 (4)	−0.0005 (3)	0.0004 (3)	0.0015 (3)
C7	0.0124 (5)	0.0036 (3)	0.0120 (4)	0.0014 (3)	0.0016 (3)	0.0018 (3)
C8	0.0153 (5)	0.0090 (4)	0.0126 (4)	0.0002 (3)	−0.0017 (3)	0.0004 (3)
N1	0.0173 (4)	0.0034 (3)	0.0142 (4)	−0.0007 (3)	−0.0003 (3)	0.0023 (3)
O1	0.0117 (4)	0.0076 (3)	0.0195 (3)	0.0000 (2)	0.0012 (3)	0.0084 (2)
O2	0.0140 (4)	0.0065 (3)	0.0191 (3)	0.0005 (2)	−0.0022 (3)	0.0045 (2)
O3	0.0543 (7)	0.0053 (3)	0.0411 (5)	0.0009 (4)	0.0251 (5)	−0.0028 (3)
O4	0.0309 (5)	0.0104 (4)	0.0477 (6)	−0.0020 (3)	0.0225 (5)	0.0052 (4)
F1	0.0228 (4)	0.0162 (3)	0.0232 (3)	0.0054 (3)	−0.0095 (3)	0.0030 (2)
F2	0.0197 (4)	0.0179 (3)	0.0247 (3)	−0.0100 (3)	−0.0065 (3)	0.0067 (3)
F3	0.0267 (4)	0.0320 (4)	0.0187 (3)	0.0079 (3)	−0.0028 (3)	−0.0137 (3)
Cl1	0.01275 (14)	0.00512 (10)	0.01442 (11)	−0.00170 (8)	−0.00296 (8)	0.00074 (7)

### 2-Chloro-3-nitro-5-(trifluoromethyl)benzoic acid (1) Geometric parameters (Å, º)

**Table:**

C1—C6	1.3957 (15)	C5—C8	1.5013 (15)
C1—C2	1.4054 (13)	C6—H6	0.9500
C1—C7	1.5026 (13)	C7—O2	1.2170 (14)
C2—C3	1.3937 (13)	C7—O1	1.3170 (12)
C2—Cl1	1.7148 (12)	C8—F2	1.3318 (13)
C3—C4	1.3765 (15)	C8—F3	1.3384 (13)
C3—N1	1.4733 (13)	C8—F1	1.3451 (13)
C4—C5	1.3916 (13)	N1—O3	1.2105 (13)
C4—H4	0.9500	N1—O4	1.2153 (13)
C5—C6	1.3900 (13)	O1—H1	0.86 (2)
			
C6—C1—C2	119.12 (9)	C5—C6—H6	119.5
C6—C1—C7	118.16 (8)	C1—C6—H6	119.5
C2—C1—C7	122.67 (9)	O2—C7—O1	124.60 (9)
C3—C2—C1	118.06 (9)	O2—C7—C1	123.69 (9)
C3—C2—Cl1	117.94 (7)	O1—C7—C1	111.69 (9)
C1—C2—Cl1	124.00 (8)	F2—C8—F3	107.89 (9)
C4—C3—C2	123.52 (8)	F2—C8—F1	106.91 (9)
C4—C3—N1	117.66 (9)	F3—C8—F1	105.88 (9)
C2—C3—N1	118.82 (9)	F2—C8—C5	112.71 (8)
C3—C4—C5	117.72 (9)	F3—C8—C5	111.35 (9)
C3—C4—H4	121.1	F1—C8—C5	111.73 (8)
C5—C4—H4	121.1	O3—N1—O4	125.01 (10)
C6—C5—C4	120.63 (9)	O3—N1—C3	117.49 (9)
C6—C5—C8	120.31 (8)	O4—N1—C3	117.50 (9)
C4—C5—C8	118.95 (9)	C7—O1—H1	109.2 (15)
C5—C6—C1	120.93 (9)		
			
C6—C1—C2—C3	1.05 (14)	C7—C1—C6—C5	−177.27 (9)
C7—C1—C2—C3	178.55 (8)	C6—C1—C7—O2	155.57 (10)
C6—C1—C2—Cl1	−179.86 (7)	C2—C1—C7—O2	−21.95 (15)
C7—C1—C2—Cl1	−2.36 (14)	C6—C1—C7—O1	−22.94 (12)
C1—C2—C3—C4	−1.35 (15)	C2—C1—C7—O1	159.54 (9)
Cl1—C2—C3—C4	179.51 (8)	C6—C5—C8—F2	31.69 (14)
C1—C2—C3—N1	179.50 (8)	C4—C5—C8—F2	−152.19 (10)
Cl1—C2—C3—N1	0.36 (12)	C6—C5—C8—F3	−89.73 (12)
C2—C3—C4—C5	0.19 (15)	C4—C5—C8—F3	86.40 (12)
N1—C3—C4—C5	179.35 (9)	C6—C5—C8—F1	152.10 (9)
C3—C4—C5—C6	1.26 (15)	C4—C5—C8—F1	−31.77 (13)
C3—C4—C5—C8	−174.85 (9)	C4—C3—N1—O3	94.69 (13)
C4—C5—C6—C1	−1.54 (15)	C2—C3—N1—O3	−86.11 (13)
C8—C5—C6—C1	174.52 (9)	C4—C3—N1—O4	−85.22 (13)
C2—C1—C6—C5	0.34 (14)	C2—C3—N1—O4	93.98 (12)

### 2-Chloro-3-nitro-5-(trifluoromethyl)benzoic acid (1) Hydrogen-bond geometry (Å, º)

**Table:**

*D*—H···*A*	*D*—H	H···*A*	*D*···*A*	*D*—H···*A*
O1—H1···O2^i^	0.86 (2)	1.83 (2)	2.6891 (13)	176 (2)
C4—H4···F3^ii^	0.95	2.66	3.5767 (16)	161
C6—H6···O3^iii^	0.95	2.66	3.5410 (19)	154
C6—H6···O4^iii^	0.95	2.62	3.5019 (17)	154

Symmetry codes: (i) −*x*+2, −*y*+1, −*z*+1; (ii) −*x*+1, −*y*+2, −*z*; (iii) *x*−1, *y*−1, *z*.

### 2-Chloro-3-nitro-5-(trifluoromethyl)benzamide (2) Crystal data

**Table:**

C_8_H_4_ClF_3_N_2_O_3_	*F*(000) = 1072
*M**_r_* = 268.58	*D*_x_ = 1.775 Mg m^−^^3^
Monoclinic, *P*2_1_/*c*	Mo *K*α radiation, λ = 0.71073 Å
*a* = 8.3012 (12) Å	Cell parameters from 8332 reflections
*b* = 28.230 (4) Å	θ = 2.7–32.0°
*c* = 9.1522 (14) Å	µ = 0.42 mm^−^^1^
β = 110.424 (3)°	*T* = 100 K
*V* = 2009.9 (5) Å^3^	Prism, colourless
*Z* = 8	0.05 × 0.02 × 0.01 mm

### 2-Chloro-3-nitro-5-(trifluoromethyl)benzamide (2) Data collection

**Table:**

Bruker Kappa Mach3 APEXII diffractometer	8316 independent reflections
Radiation source: microfocus X-ray tube	5593 reflections with *I* > 2σ(*I*)
Incoatec Helios mirrors monochromator	*R*_int_ = 0.080
Detector resolution: 66.67 pixels mm^-1^	θ_max_ = 34.3°, θ_min_ = 1.4°
φ– and ω–scans	*h* = −13→13
Absorption correction: gaussian (SADABS; Bruker, 2012)	*k* = −44→43
*T*_min_ = 0.989, *T*_max_ = 0.996	*l* = −14→14
64252 measured reflections	

### 2-Chloro-3-nitro-5-(trifluoromethyl)benzamide (2) Refinement

**Table:**

Refinement on *F*^2^	Primary atom site location: dual
Least-squares matrix: full	Secondary atom site location: difference Fourier map
*R*[*F*^2^ > 2σ(*F*^2^)] = 0.056	Hydrogen site location: mixed
*wR*(*F*^2^) = 0.136	H atoms treated by a mixture of independent and constrained refinement
*S* = 1.04	*w* = 1/[σ^2^(*F*_o_^2^) + (0.047*P*)^2^ + 2.2564*P*] where *P* = (*F*_o_^2^ + 2*F*_c_^2^)/3
8316 reflections	(Δ/σ)_max_ < 0.001
329 parameters	Δρ_max_ = 0.79 e Å^−^^3^
121 restraints	Δρ_min_ = −0.59 e Å^−^^3^

### 2-Chloro-3-nitro-5-(trifluoromethyl)benzamide (2) Special details

**Table:**

Geometry. All esds (except the esd in the dihedral angle between two l.s. planes) are estimated using the full covariance matrix. The cell esds are taken into account individually in the estimation of esds in distances, angles and torsion angles; correlations between esds in cell parameters are only used when they are defined by crystal symmetry. An approximate (isotropic) treatment of cell esds is used for estimating esds involving l.s. planes.

### 2-Chloro-3-nitro-5-(trifluoromethyl)benzamide (2) Fractional atomic coordinates and isotropic or equivalent isotropic displacement parameters (Å^2^)

**Table:**

	*x*	*y*	*z*	*U*_iso_*/*U*_eq_	Occ. (<1)
C1_1	0.6080 (2)	0.40838 (6)	0.4317 (2)	0.0121 (3)	
C2_1	0.4418 (2)	0.41062 (6)	0.4365 (2)	0.0132 (3)	
C3_1	0.3904 (2)	0.37581 (7)	0.5183 (2)	0.0143 (3)	
C4_1	0.4966 (2)	0.33920 (7)	0.5936 (2)	0.0142 (3)	
H4_1	0.459003	0.316214	0.650464	0.017*	
C5_1	0.6590 (2)	0.33663 (6)	0.5847 (2)	0.0125 (3)	
C6_1	0.7155 (2)	0.37105 (6)	0.5056 (2)	0.0121 (3)	
H6_1	0.828258	0.369169	0.501743	0.015*	
C7_1	0.6725 (2)	0.44400 (6)	0.3435 (2)	0.0132 (3)	
C8_1	0.7697 (2)	0.29469 (7)	0.6533 (2)	0.0164 (3)	
N1_1	0.82735 (19)	0.46157 (6)	0.41884 (19)	0.0161 (3)	
H1A_1	0.872 (3)	0.4820 (8)	0.370 (3)	0.019*	
H1B_1	0.882 (3)	0.4553 (9)	0.519 (2)	0.019*	
N2_1	0.2167 (2)	0.37541 (6)	0.5271 (2)	0.0188 (3)	
O1_1	0.58652 (17)	0.45467 (5)	0.20742 (16)	0.0211 (3)	
O2_1	0.09471 (19)	0.38169 (7)	0.4067 (2)	0.0343 (4)	
O3_1	0.2059 (2)	0.36737 (6)	0.6541 (2)	0.0299 (4)	
F1_1	0.74246 (19)	0.25946 (5)	0.55044 (16)	0.0323 (3)	
F2_1	0.93677 (16)	0.30500 (5)	0.70284 (18)	0.0316 (3)	
F3_1	0.73792 (18)	0.27697 (5)	0.77582 (15)	0.0276 (3)	
Cl1_1	0.30947 (6)	0.45781 (2)	0.35459 (6)	0.02245 (11)	
C1_2	0.9002 (2)	0.59541 (6)	0.0619 (2)	0.0114 (3)	
C2_2	1.0602 (2)	0.59679 (6)	0.0421 (2)	0.0129 (3)	
C3_2	1.0995 (2)	0.63574 (7)	−0.0321 (2)	0.0138 (3)	
C4_2	0.9863 (2)	0.67303 (7)	−0.0875 (2)	0.0155 (3)	
H4_2	1.014872	0.698891	−0.140342	0.019*	
C5_2	0.8306 (2)	0.67163 (7)	−0.0641 (2)	0.0152 (3)	
C6_2	0.7871 (2)	0.63314 (6)	0.0082 (2)	0.0133 (3)	
H6_2	0.678569	0.632474	0.021372	0.016*	
C7_2	0.8488 (2)	0.55491 (6)	0.1431 (2)	0.0123 (3)	
C8_2	0.7080 (3)	0.71245 (8)	−0.1177 (3)	0.0236 (4)	
N1_2	0.68823 (19)	0.54002 (6)	0.07712 (18)	0.0151 (3)	
H1A_2	0.655 (3)	0.5167 (7)	0.121 (3)	0.018*	
H1B_2	0.620 (3)	0.5499 (8)	−0.015 (2)	0.018*	
N2_2	1.2661 (2)	0.63984 (6)	−0.05424 (19)	0.0168 (3)	
O1_2	0.95089 (17)	0.53776 (5)	0.26449 (16)	0.0190 (3)	
O2_2	1.39629 (17)	0.63141 (6)	0.05787 (18)	0.0252 (3)	
O3_2	1.26362 (19)	0.65271 (6)	−0.18276 (18)	0.0250 (3)	
F1_2	0.7834 (3)	0.75213 (7)	−0.1279 (4)	0.0713 (10)	0.876 (3)
F2_2	0.6181 (3)	0.72010 (7)	−0.0243 (2)	0.0392 (5)	0.876 (3)
F3_2	0.5873 (3)	0.70422 (8)	−0.2565 (2)	0.0544 (7)	0.876 (3)
F1'_2	0.5683 (15)	0.7094 (5)	−0.100 (2)	0.0713 (10)	0.124 (3)
F2'_2	0.6950 (18)	0.7262 (5)	−0.2565 (12)	0.0392 (5)	0.124 (3)
F3'_2	0.7907 (17)	0.7497 (4)	−0.0237 (15)	0.0544 (7)	0.124 (3)
Cl1_2	1.19856 (6)	0.54955 (2)	0.09572 (7)	0.02395 (11)	

### 2-Chloro-3-nitro-5-(trifluoromethyl)benzamide (2) Atomic displacement parameters (Å^2^)

**Table:**

	*U*^11^	*U*^22^	*U*^33^	*U*^12^	*U*^13^	*U*^23^
C1_1	0.0101 (7)	0.0156 (8)	0.0101 (7)	−0.0009 (6)	0.0028 (6)	0.0013 (6)
C2_1	0.0106 (7)	0.0147 (8)	0.0129 (8)	0.0023 (6)	0.0025 (6)	0.0004 (6)
C3_1	0.0098 (7)	0.0192 (9)	0.0149 (8)	−0.0008 (6)	0.0056 (6)	−0.0029 (6)
C4_1	0.0145 (7)	0.0159 (8)	0.0136 (8)	−0.0029 (6)	0.0067 (6)	−0.0001 (6)
C5_1	0.0125 (7)	0.0144 (8)	0.0112 (7)	0.0003 (6)	0.0047 (6)	0.0006 (6)
C6_1	0.0096 (6)	0.0169 (8)	0.0100 (7)	−0.0002 (6)	0.0036 (5)	0.0014 (6)
C7_1	0.0114 (7)	0.0148 (8)	0.0117 (7)	−0.0008 (6)	0.0021 (6)	0.0018 (6)
C8_1	0.0180 (8)	0.0172 (9)	0.0132 (8)	0.0019 (6)	0.0044 (6)	0.0027 (6)
N1_1	0.0121 (6)	0.0217 (8)	0.0110 (7)	−0.0047 (6)	−0.0002 (5)	0.0043 (6)
N2_1	0.0125 (7)	0.0239 (8)	0.0230 (8)	−0.0019 (6)	0.0098 (6)	−0.0043 (7)
O1_1	0.0169 (6)	0.0276 (8)	0.0127 (6)	−0.0077 (5)	−0.0025 (5)	0.0069 (5)
O2_1	0.0109 (6)	0.0605 (12)	0.0298 (9)	−0.0005 (7)	0.0050 (6)	−0.0026 (8)
O3_1	0.0263 (8)	0.0415 (10)	0.0304 (9)	−0.0029 (7)	0.0205 (7)	−0.0020 (7)
F1_1	0.0434 (8)	0.0258 (7)	0.0223 (7)	0.0154 (6)	0.0047 (6)	−0.0048 (5)
F2_1	0.0139 (5)	0.0323 (7)	0.0436 (8)	0.0049 (5)	0.0037 (5)	0.0153 (6)
F3_1	0.0354 (7)	0.0270 (7)	0.0234 (6)	0.0068 (5)	0.0142 (6)	0.0130 (5)
Cl1_1	0.0176 (2)	0.0227 (2)	0.0252 (2)	0.00890 (17)	0.00511 (17)	0.00489 (19)
C1_2	0.0086 (6)	0.0147 (8)	0.0101 (7)	−0.0017 (5)	0.0024 (5)	−0.0005 (6)
C2_2	0.0095 (7)	0.0151 (8)	0.0134 (7)	0.0007 (6)	0.0031 (6)	−0.0004 (6)
C3_2	0.0089 (7)	0.0192 (8)	0.0136 (8)	−0.0023 (6)	0.0045 (6)	−0.0019 (6)
C4_2	0.0146 (7)	0.0167 (8)	0.0151 (8)	−0.0037 (6)	0.0048 (6)	0.0016 (6)
C5_2	0.0109 (7)	0.0162 (8)	0.0168 (8)	0.0001 (6)	0.0029 (6)	0.0019 (7)
C6_2	0.0083 (6)	0.0159 (8)	0.0146 (8)	−0.0003 (6)	0.0026 (6)	0.0005 (6)
C7_2	0.0102 (7)	0.0146 (8)	0.0107 (7)	−0.0016 (6)	0.0019 (6)	0.0011 (6)
C8_2	0.0194 (9)	0.0207 (10)	0.0305 (11)	0.0040 (7)	0.0086 (8)	0.0068 (8)
N1_2	0.0116 (6)	0.0181 (8)	0.0125 (7)	−0.0037 (5)	0.0002 (5)	0.0045 (6)
N2_2	0.0142 (7)	0.0198 (8)	0.0191 (8)	−0.0033 (6)	0.0093 (6)	−0.0028 (6)
O1_2	0.0141 (6)	0.0241 (7)	0.0133 (6)	−0.0044 (5)	−0.0021 (5)	0.0067 (5)
O2_2	0.0105 (6)	0.0394 (9)	0.0249 (8)	−0.0017 (6)	0.0051 (5)	−0.0017 (7)
O3_2	0.0236 (7)	0.0350 (9)	0.0215 (7)	−0.0059 (6)	0.0143 (6)	0.0006 (6)
F1_2	0.0288 (9)	0.0234 (9)	0.168 (3)	0.0095 (7)	0.0429 (14)	0.0400 (13)
F2_2	0.0442 (10)	0.0398 (11)	0.0407 (10)	0.0245 (8)	0.0235 (9)	0.0100 (8)
F3_2	0.0489 (13)	0.0597 (14)	0.0341 (10)	0.0336 (11)	−0.0113 (8)	−0.0033 (9)
F1'_2	0.0288 (9)	0.0234 (9)	0.168 (3)	0.0095 (7)	0.0429 (14)	0.0400 (13)
F2'_2	0.0442 (10)	0.0398 (11)	0.0407 (10)	0.0245 (8)	0.0235 (9)	0.0100 (8)
F3'_2	0.0489 (13)	0.0597 (14)	0.0341 (10)	0.0336 (11)	−0.0113 (8)	−0.0033 (9)
Cl1_2	0.0173 (2)	0.0205 (2)	0.0365 (3)	0.00767 (17)	0.01266 (19)	0.0078 (2)

### 2-Chloro-3-nitro-5-(trifluoromethyl)benzamide (2) Geometric parameters (Å, º)

**Table:**

C1_1—C6_1	1.394 (2)	C1_2—C7_2	1.505 (2)
C1_1—C2_1	1.397 (2)	C2_2—C3_2	1.390 (3)
C1_1—C7_1	1.500 (2)	C2_2—Cl1_2	1.7163 (18)
C2_1—C3_1	1.390 (3)	C3_2—C4_2	1.384 (3)
C2_1—Cl1_1	1.7229 (18)	C3_2—N2_2	1.470 (2)
C3_1—C4_1	1.377 (3)	C4_2—C5_2	1.383 (2)
C3_1—N2_1	1.471 (2)	C4_2—H4_2	0.9500
C4_1—C5_1	1.381 (2)	C5_2—C6_2	1.384 (3)
C4_1—H4_1	0.9500	C5_2—C8_2	1.502 (3)
C5_1—C6_1	1.388 (2)	C6_2—H6_2	0.9500
C5_1—C8_1	1.496 (3)	C7_2—O1_2	1.236 (2)
C6_1—H6_1	0.9500	C7_2—N1_2	1.325 (2)
C7_1—O1_1	1.237 (2)	C8_2—F1'_2	1.227 (9)
C7_1—N1_1	1.325 (2)	C8_2—F2'_2	1.297 (9)
C8_1—F2_1	1.332 (2)	C8_2—F1_2	1.302 (3)
C8_1—F1_1	1.333 (2)	C8_2—F2_2	1.334 (3)
C8_1—F3_1	1.336 (2)	C8_2—F3_2	1.335 (3)
N1_1—H1A_1	0.887 (16)	C8_2—F3'_2	1.381 (10)
N1_1—H1B_1	0.886 (16)	N1_2—H1A_2	0.863 (16)
N2_1—O3_1	1.218 (2)	N1_2—H1B_2	0.884 (16)
N2_1—O2_1	1.222 (2)	N2_2—O3_2	1.224 (2)
C1_2—C6_2	1.391 (2)	N2_2—O2_2	1.226 (2)
C1_2—C2_2	1.402 (2)		
			
C6_1—C1_1—C2_1	119.42 (16)	C3_2—C2_2—C1_2	118.66 (16)
C6_1—C1_1—C7_1	118.65 (14)	C3_2—C2_2—Cl1_2	120.43 (13)
C2_1—C1_1—C7_1	121.88 (16)	C1_2—C2_2—Cl1_2	120.75 (14)
C3_1—C2_1—C1_1	118.51 (16)	C4_2—C3_2—C2_2	122.40 (15)
C3_1—C2_1—Cl1_1	120.79 (13)	C4_2—C3_2—N2_2	116.11 (16)
C1_1—C2_1—Cl1_1	120.53 (14)	C2_2—C3_2—N2_2	121.49 (16)
C4_1—C3_1—C2_1	122.45 (15)	C5_2—C4_2—C3_2	118.22 (17)
C4_1—C3_1—N2_1	115.82 (16)	C5_2—C4_2—H4_2	120.9
C2_1—C3_1—N2_1	121.72 (16)	C3_2—C4_2—H4_2	120.9
C3_1—C4_1—C5_1	118.54 (16)	C4_2—C5_2—C6_2	120.71 (17)
C3_1—C4_1—H4_1	120.7	C4_2—C5_2—C8_2	119.39 (17)
C5_1—C4_1—H4_1	120.7	C6_2—C5_2—C8_2	119.91 (16)
C4_1—C5_1—C6_1	120.59 (16)	C5_2—C6_2—C1_2	120.89 (15)
C4_1—C5_1—C8_1	119.07 (16)	C5_2—C6_2—H6_2	119.6
C6_1—C5_1—C8_1	120.24 (15)	C1_2—C6_2—H6_2	119.6
C5_1—C6_1—C1_1	120.45 (15)	O1_2—C7_2—N1_2	123.41 (16)
C5_1—C6_1—H6_1	119.8	O1_2—C7_2—C1_2	121.18 (15)
C1_1—C6_1—H6_1	119.8	N1_2—C7_2—C1_2	115.40 (15)
O1_1—C7_1—N1_1	123.15 (17)	F1'_2—C8_2—F2'_2	112.8 (9)
O1_1—C7_1—C1_1	121.07 (15)	F1_2—C8_2—F2_2	107.2 (2)
N1_1—C7_1—C1_1	115.74 (15)	F1_2—C8_2—F3_2	107.6 (2)
F2_1—C8_1—F1_1	107.64 (16)	F2_2—C8_2—F3_2	103.65 (19)
F2_1—C8_1—F3_1	106.43 (15)	F1'_2—C8_2—F3'_2	105.0 (9)
F1_1—C8_1—F3_1	106.19 (16)	F2'_2—C8_2—F3'_2	103.6 (8)
F2_1—C8_1—C5_1	112.58 (16)	F1'_2—C8_2—C5_2	117.9 (7)
F1_1—C8_1—C5_1	111.49 (15)	F2'_2—C8_2—C5_2	111.4 (5)
F3_1—C8_1—C5_1	112.13 (15)	F1_2—C8_2—C5_2	113.31 (18)
C7_1—N1_1—H1A_1	118.7 (16)	F2_2—C8_2—C5_2	112.42 (18)
C7_1—N1_1—H1B_1	121.3 (16)	F3_2—C8_2—C5_2	112.05 (19)
H1A_1—N1_1—H1B_1	120 (2)	F3'_2—C8_2—C5_2	104.4 (5)
O3_1—N2_1—O2_1	125.01 (17)	C7_2—N1_2—H1A_2	117.4 (16)
O3_1—N2_1—C3_1	116.95 (16)	C7_2—N1_2—H1B_2	122.9 (16)
O2_1—N2_1—C3_1	118.01 (16)	H1A_2—N1_2—H1B_2	119 (2)
C6_2—C1_2—C2_2	119.09 (16)	O3_2—N2_2—O2_2	125.16 (16)
C6_2—C1_2—C7_2	118.92 (14)	O3_2—N2_2—C3_2	117.04 (16)
C2_2—C1_2—C7_2	121.97 (15)	O2_2—N2_2—C3_2	117.77 (15)
			
C6_1—C1_1—C2_1—C3_1	1.4 (3)	C7_2—C1_2—C2_2—Cl1_2	5.6 (2)
C7_1—C1_1—C2_1—C3_1	178.83 (17)	C1_2—C2_2—C3_2—C4_2	−0.3 (3)
C6_1—C1_1—C2_1—Cl1_1	176.81 (14)	Cl1_2—C2_2—C3_2—C4_2	175.15 (15)
C7_1—C1_1—C2_1—Cl1_1	−5.8 (2)	C1_2—C2_2—C3_2—N2_2	178.95 (16)
C1_1—C2_1—C3_1—C4_1	−0.4 (3)	Cl1_2—C2_2—C3_2—N2_2	−5.6 (2)
Cl1_1—C2_1—C3_1—C4_1	−175.85 (15)	C2_2—C3_2—C4_2—C5_2	1.7 (3)
C1_1—C2_1—C3_1—N2_1	−179.22 (16)	N2_2—C3_2—C4_2—C5_2	−177.58 (16)
Cl1_1—C2_1—C3_1—N2_1	5.4 (2)	C3_2—C4_2—C5_2—C6_2	−2.2 (3)
C2_1—C3_1—C4_1—C5_1	−1.2 (3)	C3_2—C4_2—C5_2—C8_2	177.74 (18)
N2_1—C3_1—C4_1—C5_1	177.60 (16)	C4_2—C5_2—C6_2—C1_2	1.3 (3)
C3_1—C4_1—C5_1—C6_1	2.0 (3)	C8_2—C5_2—C6_2—C1_2	−178.60 (18)
C3_1—C4_1—C5_1—C8_1	−174.41 (17)	C2_2—C1_2—C6_2—C5_2	0.1 (3)
C4_1—C5_1—C6_1—C1_1	−1.0 (3)	C7_2—C1_2—C6_2—C5_2	178.55 (17)
C8_1—C5_1—C6_1—C1_1	175.30 (17)	C6_2—C1_2—C7_2—O1_2	−135.37 (19)
C2_1—C1_1—C6_1—C5_1	−0.7 (3)	C2_2—C1_2—C7_2—O1_2	43.0 (3)
C7_1—C1_1—C6_1—C5_1	−178.19 (16)	C6_2—C1_2—C7_2—N1_2	43.5 (2)
C6_1—C1_1—C7_1—O1_1	128.86 (19)	C2_2—C1_2—C7_2—N1_2	−138.08 (18)
C2_1—C1_1—C7_1—O1_1	−48.6 (3)	C4_2—C5_2—C8_2—F1'_2	177.4 (11)
C6_1—C1_1—C7_1—N1_1	−49.0 (2)	C6_2—C5_2—C8_2—F1'_2	−2.7 (11)
C2_1—C1_1—C7_1—N1_1	133.54 (19)	C4_2—C5_2—C8_2—F2'_2	44.6 (8)
C4_1—C5_1—C8_1—F2_1	−150.70 (17)	C6_2—C5_2—C8_2—F2'_2	−135.5 (8)
C6_1—C5_1—C8_1—F2_1	32.9 (2)	C4_2—C5_2—C8_2—F1_2	−24.5 (3)
C4_1—C5_1—C8_1—F1_1	88.2 (2)	C6_2—C5_2—C8_2—F1_2	155.5 (3)
C6_1—C5_1—C8_1—F1_1	−88.2 (2)	C4_2—C5_2—C8_2—F2_2	−146.3 (2)
C4_1—C5_1—C8_1—F3_1	−30.7 (2)	C6_2—C5_2—C8_2—F2_2	33.7 (3)
C6_1—C5_1—C8_1—F3_1	152.89 (17)	C4_2—C5_2—C8_2—F3_2	97.5 (2)
C4_1—C3_1—N2_1—O3_1	45.0 (2)	C6_2—C5_2—C8_2—F3_2	−82.6 (3)
C2_1—C3_1—N2_1—O3_1	−136.1 (2)	C4_2—C5_2—C8_2—F3'_2	−66.6 (8)
C4_1—C3_1—N2_1—O2_1	−132.8 (2)	C6_2—C5_2—C8_2—F3'_2	113.3 (8)
C2_1—C3_1—N2_1—O2_1	46.0 (3)	C4_2—C3_2—N2_2—O3_2	−45.6 (2)
C6_2—C1_2—C2_2—C3_2	−0.6 (3)	C2_2—C3_2—N2_2—O3_2	135.17 (19)
C7_2—C1_2—C2_2—C3_2	−179.02 (16)	C4_2—C3_2—N2_2—O2_2	132.50 (19)
C6_2—C1_2—C2_2—Cl1_2	−176.04 (14)	C2_2—C3_2—N2_2—O2_2	−46.8 (3)

### 2-Chloro-3-nitro-5-(trifluoromethyl)benzamide (2) Hydrogen-bond geometry (Å, º)

**Table:**

*D*—H···*A*	*D*—H	H···*A*	*D*···*A*	*D*—H···*A*
N1_1—H1*A*_1···O1_2	0.89 (2)	2.07 (2)	2.947 (2)	170 (2)
N1_1—H1*B*_1···O1_2^i^	0.89 (2)	1.99 (2)	2.840 (2)	160 (2)
N1_2—H1*A*_2···O1_1	0.86 (2)	2.08 (2)	2.940 (2)	172 (2)
N1_2—H1*B*_2···O1_1^ii^	0.88 (2)	1.99 (2)	2.804 (2)	153 (2)
C6_1—H6_1···O2_1^iii^	0.95	2.67	3.578 (2)	161
C6_2—H6_2···O2_2^iv^	0.95	2.48	3.430 (2)	179

Symmetry codes: (i) −*x*+2, −*y*+1, −*z*+1; (ii) −*x*+1, −*y*+1, −*z*; (iii) *x*+1, *y*, *z*; (iv) *x*−1, *y*, *z*.
