# Supplementary material for: 2-Chloro-3-nitro-5-(tri­fluoro­meth­yl)benzoic acid and -benzamide: structural characterization of two precursors for anti­tubercular benzo­thia­zinones
Source: Acta Crystallogr E Crystallogr Commun. 2021 Jan 19;77(Pt 2):142–7. doi: 10.1107/S2056989021000517 (PMC7869535; doi:10.1107/S2056989021000517)
Supplement: Supplementary file 4 [file e-77-00142-sup4.pdf]

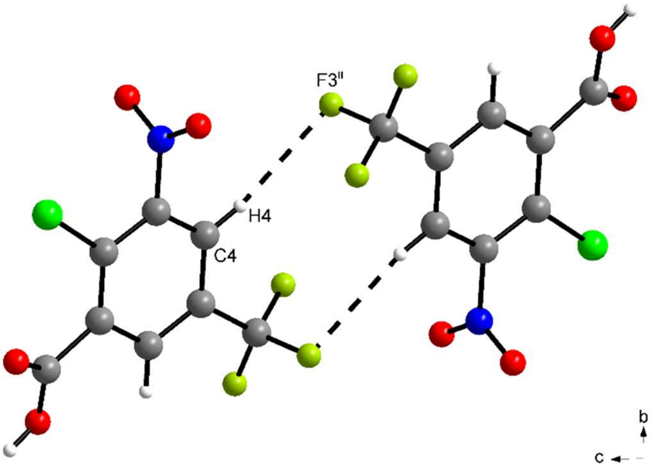

Figure S1 Part of the crystal structure of **1**, showing C—H···F (viewed along the *a* axis), represented by dashed lines. Symmetry codes: (ii) –x + 1, –y + 2, –z.

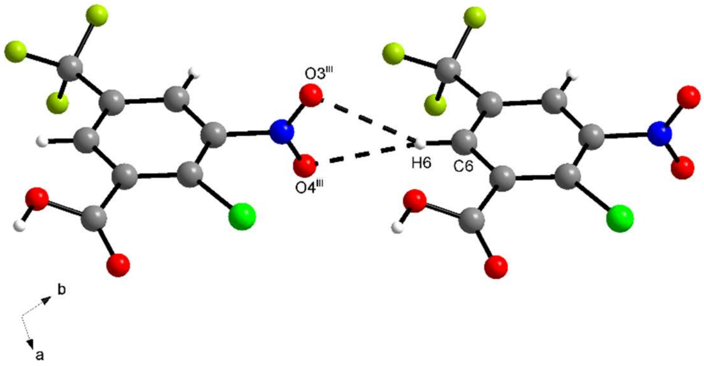

Figure S2 Part of the crystal structure of **1**, showing donating bifurcated C—H···O possible weak hydrogen bonds (viewed along the c axis), represented by dashed lines. Symmetry code: (iii) x–1, y–1, z.

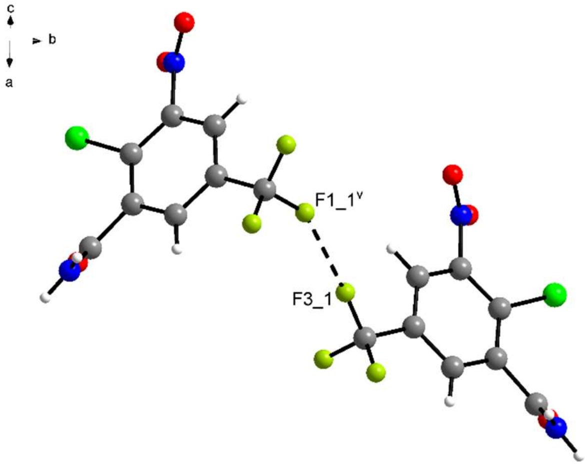

Figure S3 Part of the crystal structure of **2**, viewed along [101], showing F···F short contacts [2.704 (2) Å] between adjacent symmetry-related molecules 1. Symmetry code: (v) x, –y + 1/2, z + 1/2.

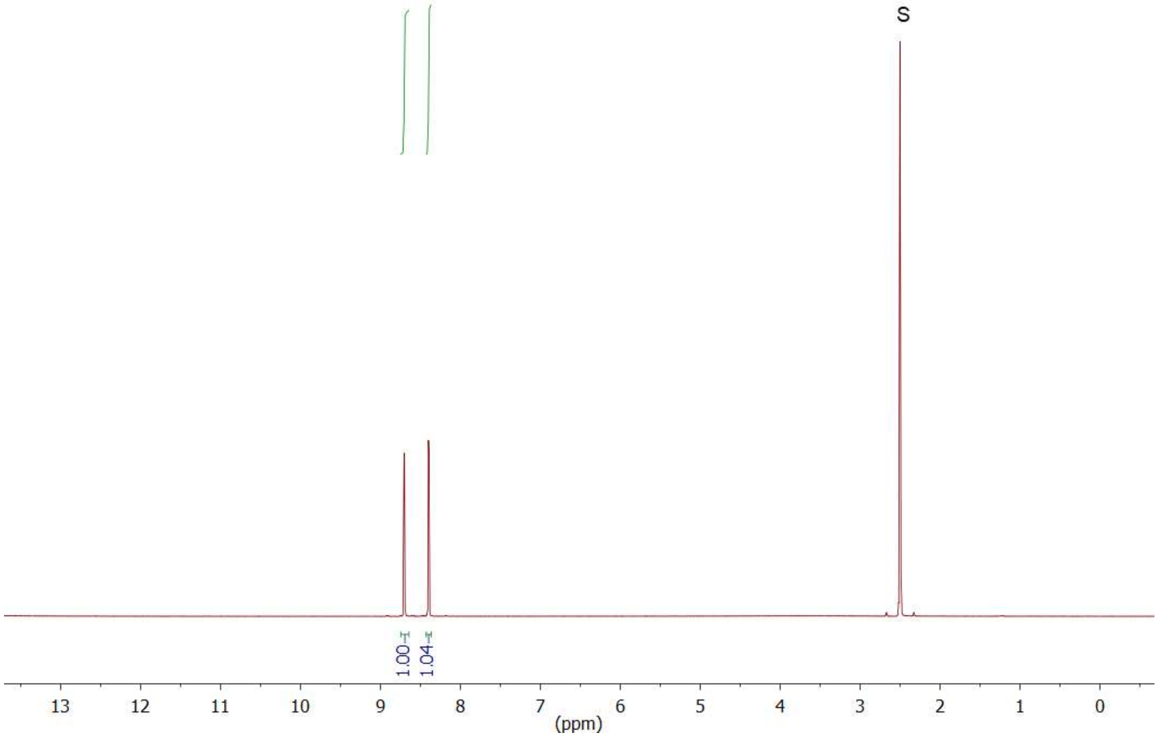

Figure S4 ^{1}H NMR spectrum of **1** in DMSO-*d6*. S denotes the residual solvent signal.

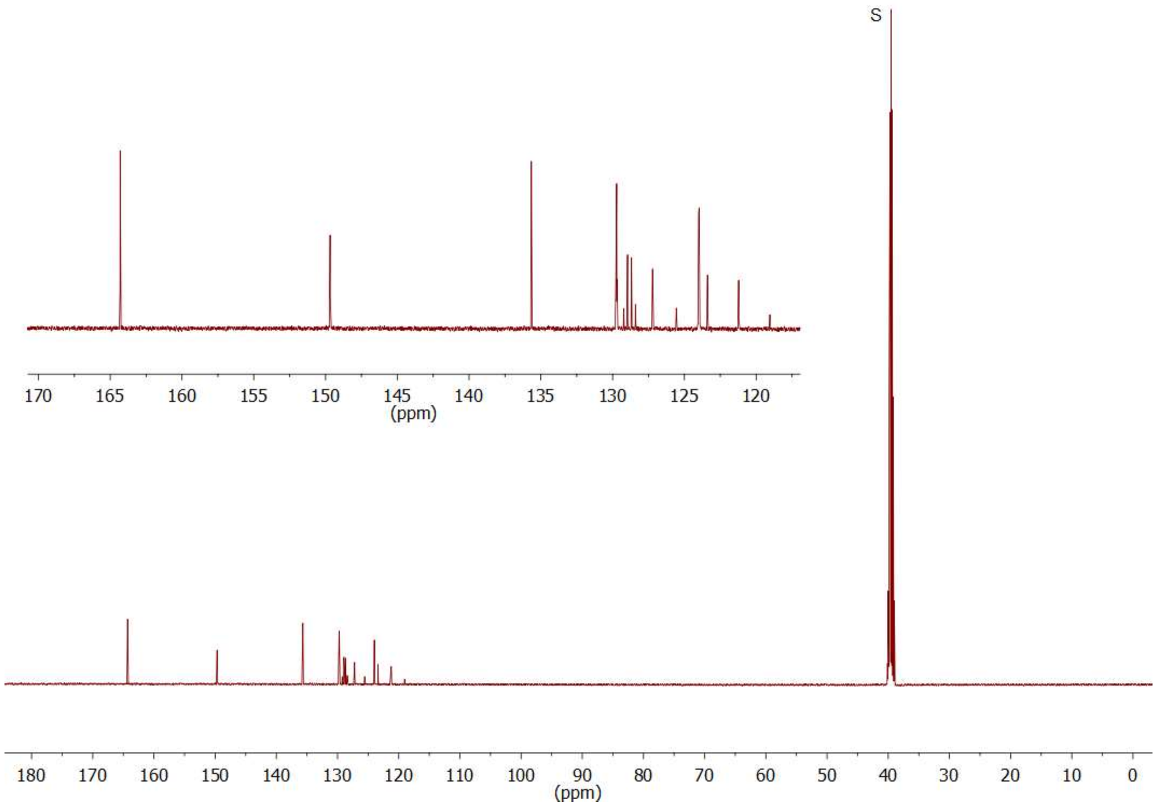

Figure S5 $^{13}C\{^{1}H\}$ NMR spectrum of **1** in DMSO-*d6*. S denotes the residual solvent signal. The inset shows the aromatic region.

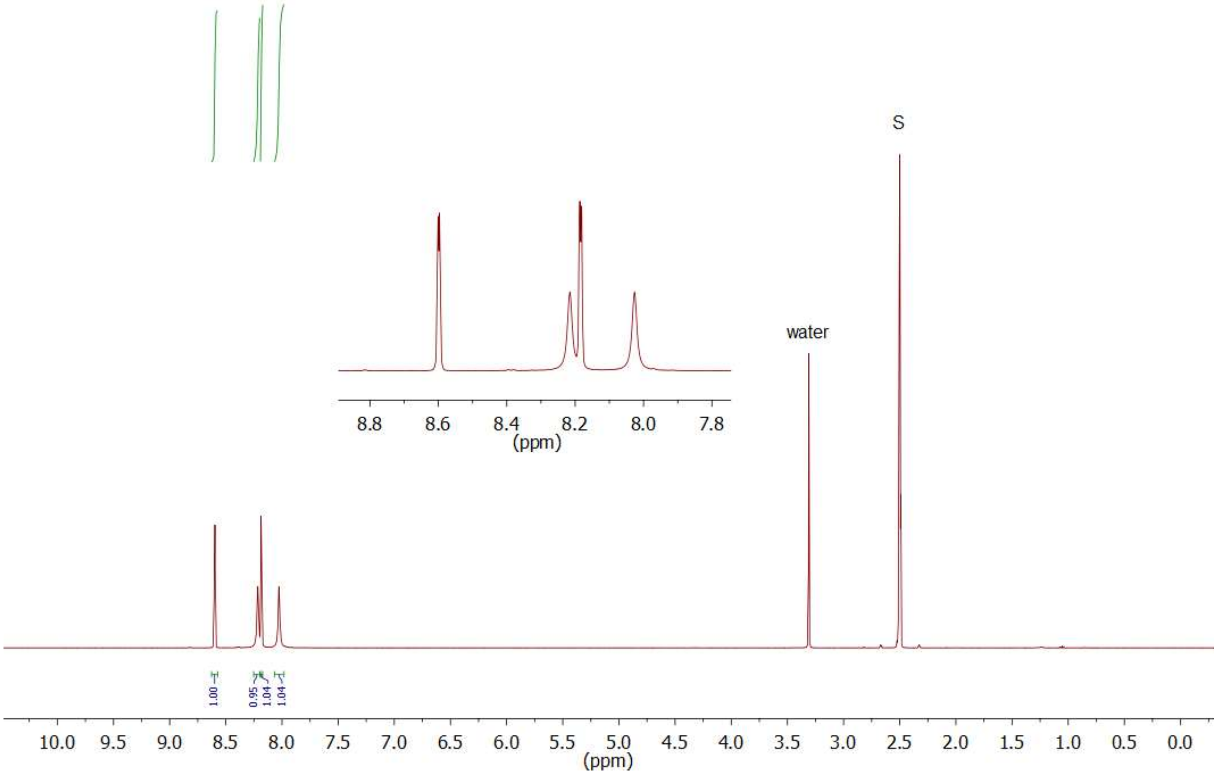

Figure S6 ^{1}H NMR spectrum of **2** in DMSO-*d*6. S denotes the residual solvent signal. The inset shows the aromatic region.

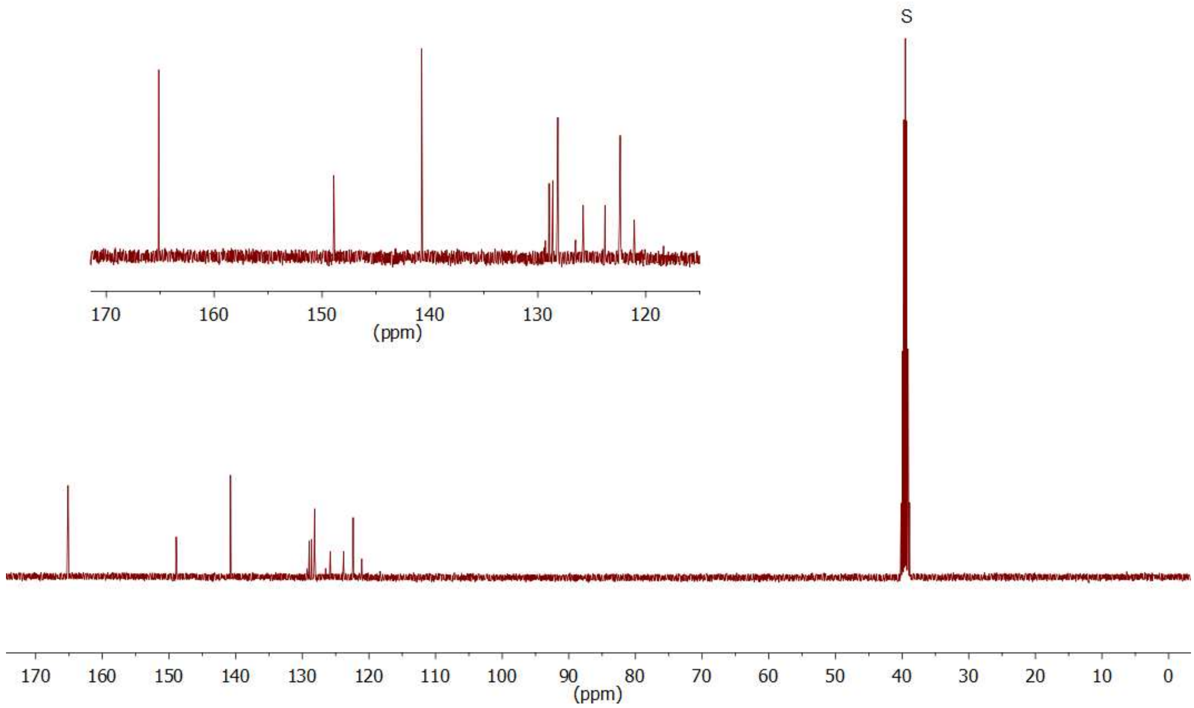

Figure S7 $^{13}C\{^{1}H\}$ NMR spectrum of **2** in DMSO-*d*6. S denotes the residual solvent signal. The inset shows the aromatic region.

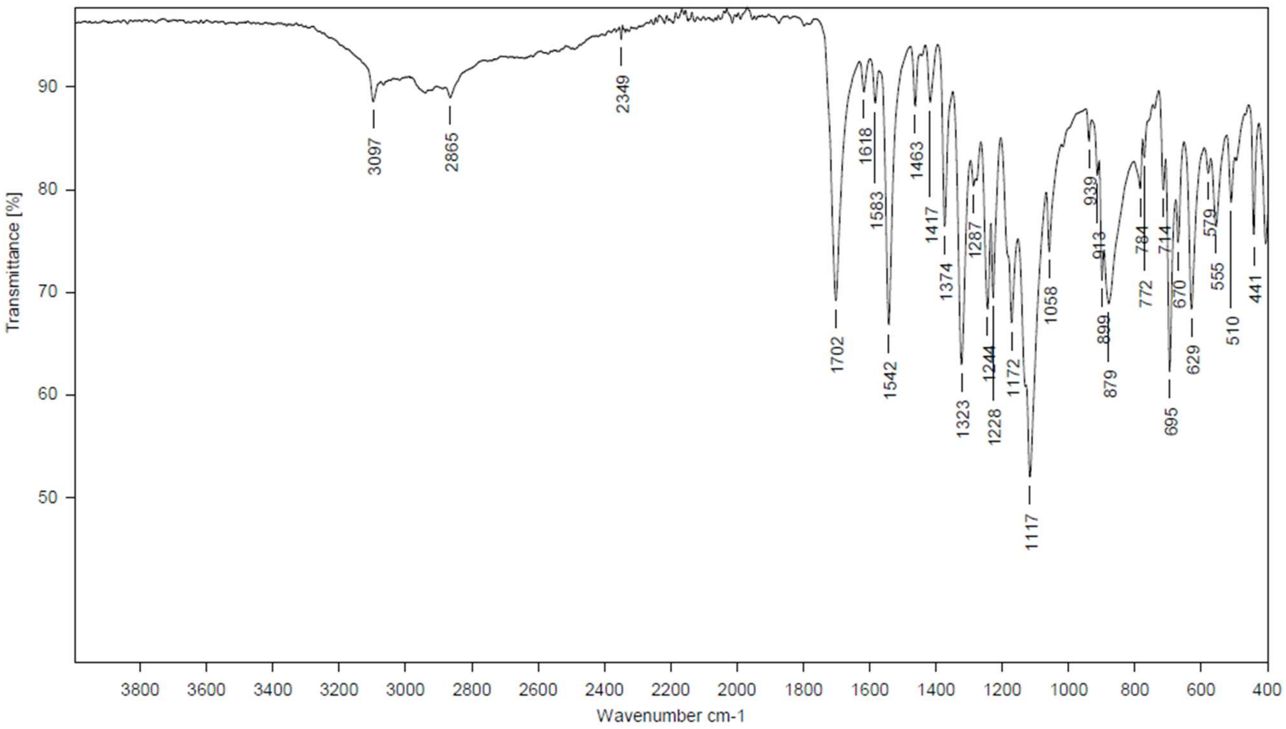

Figure S8 Solid-state ATR-FT-IR spectrum of **1** at a resolution of 4 cm^{-1} (32 scans).

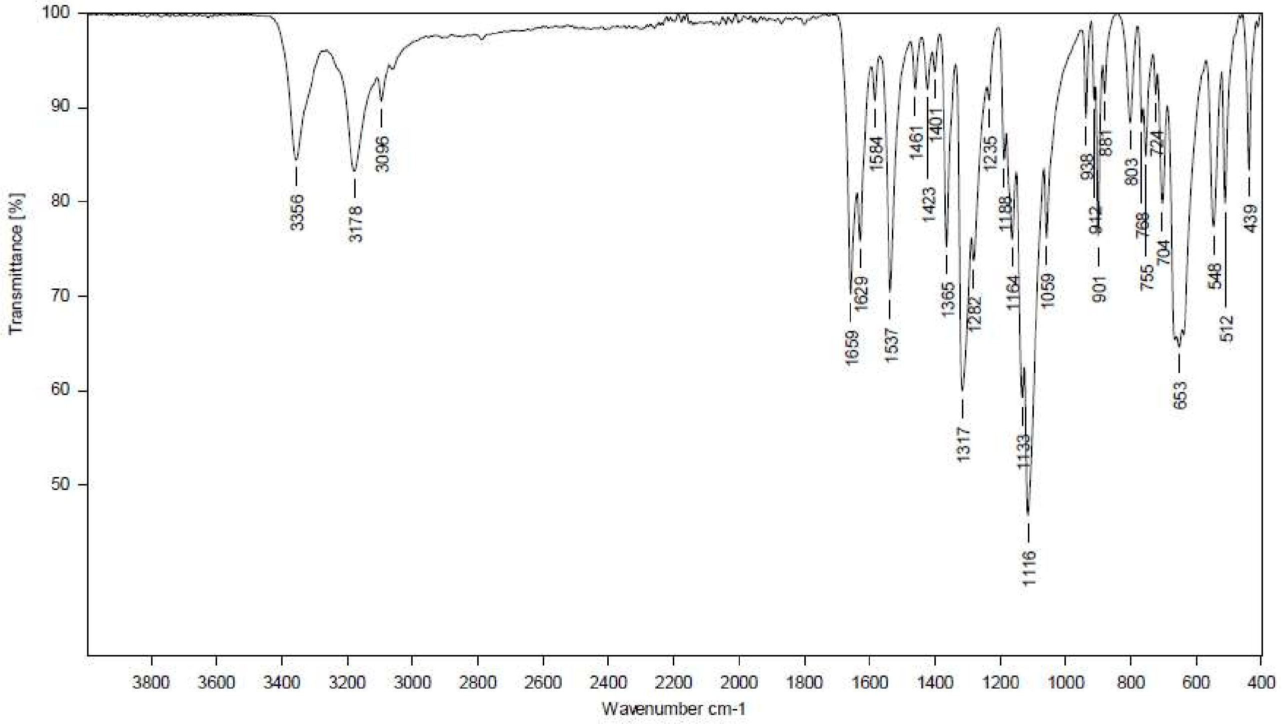

Figure S9 Solid-state ATR-FT-IR spectrum of **2** at a resolution of 4 cm^{-1} (32 scans).

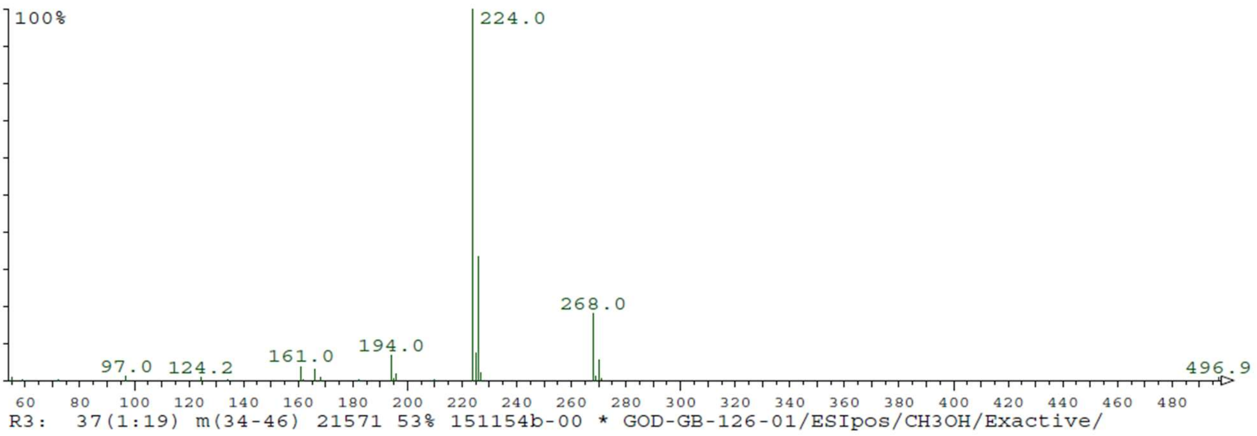

Figure S10 ESI⁻ mass spectrum of **1** in methanol.

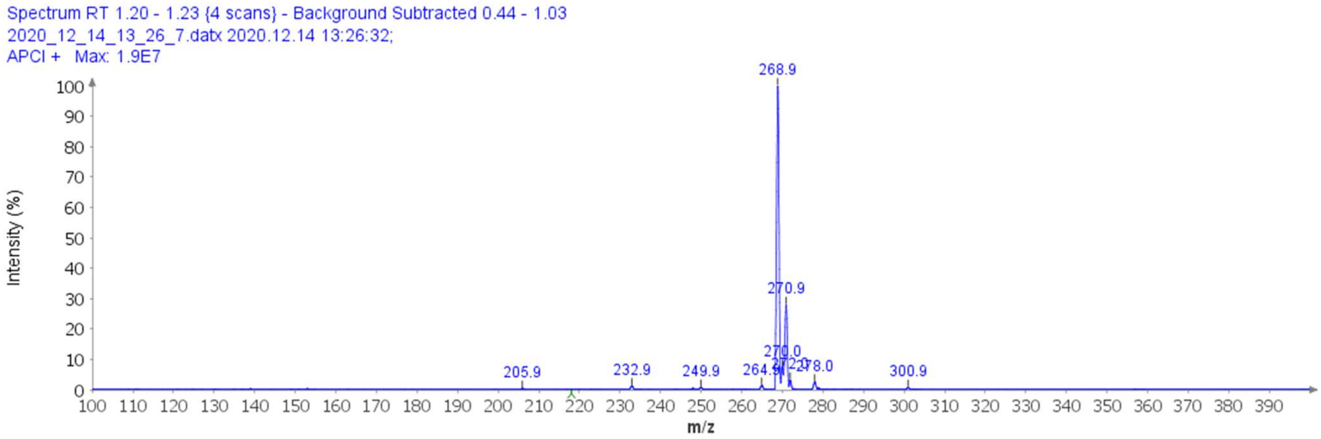

Figure S11 APCI$^+$ mass spectrum of **2** in methanol.
